# Restoring autophagic function: a case for type 2 diabetes mellitus drug repurposing in Parkinson’s disease

**DOI:** 10.3389/fnins.2023.1244022

**Published:** 2023-11-03

**Authors:** Marco Greco, Anas Munir, Debora Musarò, Chiara Coppola, Michele Maffia

**Affiliations:** ^1^Department of Biological and Environmental Science and Technology, University of Salento, Lecce, Italy; ^2^Department of Mathematics and Physics “E. De Giorgi”, University of Salento, Lecce, Italy

**Keywords:** type 2 diabetes mellitus, Parkinson’s disease, alpha-synuclein, islet amyloid peptide protein, insulin-resistance, autophagy, hyperglycemia

## Abstract

Parkinson’s disease (PD) is a predominantly idiopathic pathological condition characterized by protein aggregation phenomena, whose main component is alpha-synuclein. Although the main risk factor is ageing, numerous evidence points to the role of type 2 diabetes mellitus (T2DM) as an etiological factor. Systemic alterations classically associated with T2DM like insulin resistance and hyperglycemia modify biological processes such as autophagy and mitochondrial homeostasis. High glucose levels also compromise protein stability through the formation of advanced glycation end products, promoting protein aggregation processes. The ability of antidiabetic drugs to act on pathways impaired in both T2DM and PD suggests that they may represent a useful tool to counteract the neurodegeneration process. Several clinical studies now in advanced stages are looking for confirmation in this regard.

## Graphical abstract

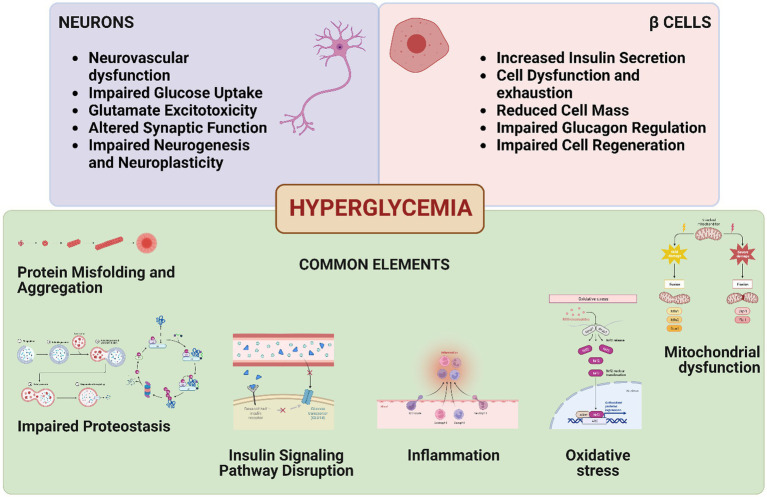

This figure represents the shared elements between neurodegeneration and β-cell dysfunction, linked through the overarching condition of hyperglycemia.

## 1. Introduction

Parkinson’s disease (PD) is an irreversible and progressive neurological condition that affects 8.5 million people worldwide (World Health Organization, 2022), with an incidence expected to double by 2040 (Dorsey and Bloem, 2018). PD classically involves midbrain structures, causing the depletion of the residing dopaminergic neuronal population and a predominantly motor symptomatology. The disease belongs to the class of proteinopathies and, apart from 5% of genetically linked cases, it is considered idiopathic (Reed et al., 2019).

Proteinopathies are chronic conditions characterized by an imbalance between protein synthesis and degradation (Bayer, 2015). This imbalance, which is caused by ageing, mitochondrial dysfunction, oxidative stress, inflammation and alterations in post-translational processes (PTMs), is responsible for protein aggregation and Lewy bodies formation at the neuronal level in PD. Type 2 diabetes mellitus (T2DM) is another proteinopathy with an alarming prevalence rate of 536 million worldwide, characterized by peripheral insulin resistance, high blood glucose levels and increased insulin secretion, which in time lead to a diminishment of β-cell functionality and number (Sun et al., 2022). Furthermore, these disease symptoms when sustained cause mitochondrial dysfunction, inflammation, and protein misfolding.

An association between type 2 diabetes mellitus (T2DM) and PD has long been advocated (Sandyk, 1993), and subsequently strengthened by a better understanding of the underlying pathologically involved pathways. Numerous population-based studies have failed to unequivocally confirm the hypothesis of a causal relationship, with often contradictory conclusions requiring further investigation (Driver et al., 2008; Cereda et al., 2011, 2012; Schernhammer et al., 2011; Savica et al., 2012; Lu et al., 2014; Pagano et al., 2018). However, because of the brain requirement of insulin and the similar signaling mechanisms in the two body districts, impaired insulin release or uptake can contribute to both T2DM and PD pathogenesis. Both the conditions share pathophysiological elements like chronic inflammation, lysosomal and mitochondrial disfunction, whose molecular bases are associated with a loss in glucose metabolism (Burbulla et al., 2017; Ma et al., 2017). Moreover, insulin signaling has been shown to be involved in several mechanisms regulating apoptosis and oxidative stress prevention, with a profound role as neuroprotective agent (Duarte et al., 2008; Serhan et al., 2020; Gayen et al., 2022).

Furthermore, the dysfunction of glucose metabolism, typical of T2DM has been demonstrated as an early marker of PD (Dunn et al., 2014). Another important process that is impacted in both diseases is the cellular recycling and degradation mechanism of autophagy, which is interestingly both disturbed and contributes to the overwhelming aggregation of proteinaceous Lewy bodies and amyloid plaques in PD and T2DM, respectively (Hou et al., 2020). Moreover, mutations in autophagy-related genes (ARGs) also inherently impair the autophagic flux in both the diseases (Simón-Sánchez et al., 2009; Cui and Li, 2023). In view of this, a limited number of studies have demonstrated that the activation of pathways like the mammalian target of rapamycin (mTOR) can restore autophagy, alleviating symptoms in *in vitro* and *in vivo* models of the two diseases (Pupyshev et al., 2021; Cui and Li, 2023). Targeting autophagy, therefore, presents a lucrative drug target common to both disorders.

## 2. Protein aggregation as a two-way connection

It has long been known, thanks to the pioneering observations of Polymeropoulos and co-workers first, Spillantini and colleagues later, that Lewy bodies in PD, initially localized in the *substantia nigra pars compacta*, are predominantly composed of alpha-synuclein (aS) protein (Polymeropoulos et al., 1997; Spillantini et al., 1997). It was subsequently discovered by Fujiwara et al. (2002) that aggregates were mainly composed of the phospho-Ser129 form of the protein (Fujiwara et al., 2002). The loss of function of the protein because of its aggregation has several consequences in dopaminergic neurons, it being involved in the modulation of neurotransmitter synthesis and, once incorporated into vesicles, also in their transport and release (Abeliovich et al., 2000; Cabin et al., 2002). Interactors of aS include tyrosine 3-monooxygenase (TH), the rate-limiting dopamine biosynthetic enzyme (Perez et al., 2002), dopamine decarboxylase (Tehranian et al., 2006), protein phosphatase 2A (PP2A) (Qu et al., 2018), vesicle membranes (Jao et al., 2004) and SNARE proteins (Burré et al., 2014). An important but still poorly understood interactor of aS is the Kir6.2 (internal rectifier potassium channel 6.2) subunit of the ATP-sensitive potassium (KATP) channel and its associated sulfonylurea receptor 1 (SUR1). This interaction modulates dopamine release by reducing it (Vidal-Martinez et al., 2018; Choudhury et al., 2022).

A similar function for the protein was observed by Geng and colleagues in pancreatic β-cell, where it has been shown to act as a regulator of the release of insulin-containing vesicles, in response to glucose concentration (Geng et al., 2007). Aggregation is observed in these cells during T2DM; the major component of which is islet amyloid polypeptide (IAPP) (Cooper et al., 1987), although immunoreactivity for phospho-Ser129-aS has also been observed (Martinez-Valbuena et al., 2018).

The fibrillation process of aS passes through the formation of oligomeric structures which are neurotoxic formations able to pierce membranes and spread. At this point, the protein can be internalised by other cells or flow through the cerebrospinal fluid (CSF) to the bloodstream (Menéndez-González et al., 2018; Karpowicz et al., 2019). A role for aS active internalization process seems to be played by surface prion protein (PrP), expressed both at the nervous and pancreatic levels, where it modulates insulin and glucose homeostasis through metal interaction (Ashok and Singh, 2018; Jucker and Walker, 2018; De Riccardis et al., 2019). Recently, Martinez-Valbuena et al. (2021) have found the presence of cytoplasmic aggregates containing IAPP and phospho-Ser129-aS interacting with PrP in human *post-mortem* pancreatic tissues (Martinez-Valbuena et al., 2021). This offers a new perspective towards a two-way connection between the pathogenesis of T2DM and PD.

## 3. Insulin resistance and the impairment of autophagic processes

A persistent condition associated with T2DM is insulin resistance, which determines a loss of glycemic control mechanisms in peripheral organs. The brain, however, is capable of managing its own glucose requirements independently of insulin, although new evidence is casting doubt on this assumption (García-Cáceres et al., 2016; Kleinridders, 2016; Pomytkin et al., 2018). Therefore, in the brain, insulin resistance results mainly in an altered signaling pathway.

Encephalic insulin receptors are predominantly expressed in neurons, where they play a role in modulating dendritic and synaptic plasticity, being most highly expressed at these levels (Sportelli et al., 2020). The hormone is associated with a plethora of activities in the brain, such as learning, memory, cognitive functions (Craft et al., 1993), neurotransmitter release (de Bartolomeis et al., 2023), neuroprotection, neuronal proliferation, migration, and differentiation (Roger and Fellows, 1980; Schubert et al., 2003; Xu et al., 2004; Sousa-Nunes et al., 2011). These activities are all modulated by the binding of insulin to its receptors and the activation of their downstream pathways, for instance, Raf-1/MAPK/ERK or PI3K/protein kinase B (AKT) (Arnold et al., 2018). These effectors then modulate mTOR activity, promote protein synthesis activating ribosomal protein S6 kinase (S6K) (Huang et al., 2019), inhibiting the eukaryotic translation initiation factor 4E-binding protein 1 (4E-BP1) (Le Bacquer et al., 2007), while activating transcription factors like forkhead box protein O1 (FoxO1) (Tsai et al., 2003), sterol regulatory element-binding protein (SREBP) and carbohydrate-responsive element-binding protein (ChREBP) (Iizuka et al., 2004; Suzuki et al., 2010).

The mTOR protein, a central element of the mTORC1 and mTORC2 complexes, modulated by the PI3K/AKT pathway, regulates the autophagic process. Alterations in this mechanism are associated with protein aggregation and reduction in ATP/ADP and NAD^+^/NADH ratios (Heras-Sandoval et al., 2014; Steinberg and Carling, 2019; Katsyuba et al., 2020). A 2010 study found high amounts of autophagosomes with permeabilized lysosomes, indicating a shunted lysosomal-mediated autophagosome clearance contributing to disease onset in *post-mortem* PD brain (Dehay et al., 2010). This, in addition to the high levels of autophagic markers that colocalize with aS in Lewy bodies, such as microtubule-associated proteins 1A/1B light chain 3B (LC3) in its lipidated form LC3-II and ubiquitin-binding protein p62 (sequestosome-1), provides important evidence for a failure of the autophagic process during the disease (Alvarez-Erviti et al., 2010; Fellner et al., 2021).

## 4. Hyperglycemia compromises cellular proteostasis

An impaired protein degradation, although sufficient to alter cellular proteostasis, is not the only event observed during T2DM. Increased level of circulating glucose also has detrimental effects on cellular homeostasis and protein function. An engulfment of the glycolytic pathway diverts glucose towards the polyol pathway (Du et al., 2003). Then, the sustained activity of aldose reductase and sorbitol dehydrogenase causes the depletion of NADPH and NAD^+^, which impairs the capability of the cell to restore reduced glutathione (GSH) levels and causes inhibition of GAPDH (Mathebula, 2015; Lutchmansingh et al., 2018). The accumulation of triose phosphates results in methylglyoxal (MG) formation, an α-keto reactive aldehyde, and then to advanced glycation end products (AGEs) (Strom et al., 2021). Hyperglycemia also impairs the hexosamine pathway, increasing UDP-*N*-acetylglucosamine levels and altering PTMs (Du et al., 2000; Del Coco et al., 2023). As the MG detoxification is mainly mediated by the highly conserved glutathione-dependent glyoxalase I/II (GloI/II) system, a reduction in intracellular levels of GSH is toxic for the cells (Blair et al., 1979; Yadav et al., 2005). Altered glycosylation and glycation processes involve both aS and IAPP, promoting their aggregation and impairing their degradation (Vicente Miranda et al., 2017; Milordini et al., 2020).

## 5. Mitochondrial dysfunction as a fatal consequence

Insulin resistance and hyperglycemia cause mitochondria dysfunction, with ROS increase and loss in Ca^2+^ homeostasis further contributing to AGEs formation (Soejima et al., 1996). Mitochondria are responsible for cellular energy metabolism, regulating also proliferation (Diers et al., 2012), apoptosis (Wang and Youle, 2009), protein degradation (Liao et al., 2020), neurotransmitter transport, uptake and recycle (Varoqui and Erickson, 1996; Gasnier, 2000; Vos, 2010). Their dysfunction is strongly related to inflammation, which in the brain is supported by microglia through the release of pro-inflammatory cytokines.

Hyperglycemia causes alterations in the organelle morphology, triggering fission processes (Wang et al., 2012). Mitophagy, a selective form of autophagy, is responsible for the removal of damaged mitochondria; during this process, organelles are labelled for degradation by PTEN-induced putative kinase 1 (PINK1), E3 ubiquitin-protein ligase Parkin, Ubiquitin and Sequestosome-1 (Lewis and Lewis, 1915; Palikaras et al., 2018). An important role in mitochondria homeostasis is held by DJ-1 protein; it acts as a transcription factor and an antioxidant and modulates chaperones, proteases, and mitochondria activity. DJ-1 functions are modulated by its oxidative state in the cell (Canet-Avilés et al., 2004; Hao et al., 2010; Wilson, 2011; Takahashi-Niki et al., 2017).

Finally, DJ-1 has a key role in repairing MG-related damages, with a recent study demonstrating glyoxalase and weak deglyoxalase activity for the protein (Mazza et al., 2022). Mutations in PINK1, Parkin and DJ-1 are correlated with the onset of monogenic forms of PD (Guadagnolo et al., 2021). This emphasizes how mitophagy and organelle homeostasis are crucial for cell well-being, and how glycemic dyshomeostasis can induce PD.

## 6. Autophagy and hyperglycemia as new drug targets

The pharmacological approach to PD is merely symptomatic, therefore the identification of a trigger capable of inducing the pathology, and its medication, stand to represent a new complementary route of intervention. Hyperglycemia, hyperinsulinemia, hypercholesterolemia, inflammation, mitochondrial dyshomeostasis, oxidative stress, alteration of protein-degrading pathways, and aggregation are all potential targets to be exploited.

Several widely used antidiabetic and hypoglycemic drugs are currently undergoing pharmaceutical trials in various studies on cohorts of PD patients with a perspective of drug repurposing (Table 1).

**Table 1:** Clinical trials on anti-diabetic drugs in PD.

Drug	Molecule class	Anti-diabetic mechanisms of action	Clinical studies	ClinicalTrials.gov identifier	Status	Sponsor/collaborators
Metformin	Biguanides	Inhibition of hepatic gluconeogenesis (Madiraju et al., 2018; Agius et al., 2020) Restoration of peripheral insulin sensitivity and glucose uptake (Li et al., 2011; Ruegsegger et al., 2019b) Reduction of intestinal absorption of glucose (Horakova et al., 2019; Sansome et al., 2020) Modulation of GLP-1 release (Amadi et al., 2021; Lee C. B. et al., 2021) Modulation of lipid metabolism by reducing LDL cholesterol and triglycerides levels (Han et al., 2019; Hu et al., 2021; Tarry-Adkins et al., 2021; Xing et al., 2022) Anti-inflammatory and antioxidant effect (Tian et al., 2019; Luo et al., 2020)	Clinical study to evaluate the possible efficacy of metformin in patients with Parkinson’s disease	NCT05781711	Phase 2 – Recruiting	Tanta University, Tanta, Egypt
Exenatide	GLP-1 agonists	Stimulation of GLP-1 receptors (Helmstädter et al., 2020; Sterling et al., 2020; Angarita et al., 2021; Cardoso et al., 2023) Promotion of insulin secretion (Yaribeygi et al., 2019) Pro-survival effect on β-cells (Tanday et al., 2022; Zhou et al., 2022) Inhibition of glucagon release (Bai et al., 2022) Slowing of gastric emptying (Geyer et al., 2019; Shang et al., 2021; Jensterle et al., 2023) Loss of appetite (Yamamoto et al., 2003; Jensen et al., 2020; Kadouh et al., 2020) Reduction of weight (Kadouh et al., 2020; Lee S. E. et al., 2021; Arastu et al., 2022; Borlaug et al., 2023) Anti-inflammatory and antioxidant effect (Sterling et al., 2020; Martins et al., 2022; Meurot et al., 2022; Luna-Marco et al., 2023)	Exenatide once weekly over 2 years as a potential disease modifying treatment for Parkinson’s disease	NCT04232969	Phase 3 – Active, not recruiting	University College, London, United Kingdom
			Trial of exenatide for Parkinson’s disease	NCT01971242	Phase 2 – Completed	University College, London, United Kingdom
			Exenatide treatment in Parkinson’s disease	NCT04305002	Phase 2 – Active, not recruiting	Center for Neurology, Stockholm, Sweden Karolinska Institutet, Solna, Sweden
			Effects of exenatide on motor function and the brain	NCT03456687	Phase 1 – Completed	University of Florida, Gainesville, FL, United States National Institute of Neurological Disorders and Stroke (NINDS), Bethesda, MD, United States
Liraglutide			Safety and efficacy of liraglutide in Parkinson’s disease	NCT02953665	Phase 2 – Completed	Cedars Sinai Medical Center, Los Angeles, CA, United States The Cure Parkinson’s Trust, London, United Kingdom Novo Nordisk A/S, Bagsværd, Denmark
Semaglutide			GLP1R in Parkinson’s disease	NCT03659682	Phase 2 – Not yet recruiting	Oslo University Hospital, Oslo, Norway
NLY01			A clinical study of NLY01 in patient’s with early Parkinson’s disease	NCT04154072	Phase 2 – Active, not recruiting	Neuraly, Inc., Gaithersburg, MD, United States
Pioglitazone	Thiazolidinediones	PPARγ agonist effect (Soliman et al., 2019; Nazreen, 2021) Inhibition of hepatic gluconeogenesis (Rahimi et al., 2020; Asakawa et al., 2023) Promotion of fat cells maturation (Yu et al., 2023) Promotion of fat deposition into peripheral tissues (Lee et al., 2023; Liu et al., 2023) Promotion of HDL cholesterol levels increase and triglycerides levels decrease (Alam et al., 2019; Lian and Fu, 2021) Restoration of insulin sensitivity (Al-Muzafar et al., 2021; Fiorentino et al., 2021) Anti-inflammatory effect (Radwan and Hasan, 2019; Pakravan et al., 2022)	Pioglitazone in Early Parkinson’s Disease	NCT01280123	Phase 2 – Completed	University of Rochester, Rochester, NY, United States National Institute of Neurological Disorders and Stroke (NINDS), Bethesda, MD, United States Michael J. Fox Foundation for Parkinson’s Research, New York, NY, United States
Insulin	Hormone	Reduction in hematic glucose levels (Rahman et al., 2021) Increase in peripheral glucose uptake via GLUTs transporters translocation toward plasma membrane (Turner et al., 2020) Suppression of hepatic gluconeogenesis and promotion of glycogen synthesis (Rahman et al., 2021) Promotion of protein synthesis (Khalid et al., 2021) Inhibition of lipolysis and promotion of fatty acid synthesis and storage (Ahmed et al., 2021; Grabner et al., 2021) Ketone bodies formation inhibition (Garcia et al., 2020) Inhibition of catabolic processes (Batista et al., 2021)	Intranasal Insulin in Parkinson’s Disease	NCT04251585	Phase 2 – Recruiting	HealthPartners Neuroscience Center, Saint Paul, MN, United States
			Intranasal Insulin and Glutathione as an Add-On Therapy in Parkinson’s Disease	NCT05266417	Phase 2 – Recruiting	Gateway Institute for Brain Research, Fort Lauderdale, FL, United States
			Treatment of Parkinson Disease and Multiple System Atrophy Using Intranasal Insulin	NCT02064166	Phase 2 – Completed	Peter Novak, Boston, MA, United States University of Massachusetts, Worcester, MA, United States

Studies that have passed the completion date and whose status has not been verified for more than 2 years are not reported. Information extracted from clinicaltrials.gov, access date 07-10-2023.

In line with this premise, this review focuses on the compilation of a list of main drugs initially developed for treating hyperglycemia in the context of diabetes, but the activation of whose principal molecular pathways is potentially beneficial in the context of PD. The knowledge of these drugs from a pharmacokinetic and toxicological point of view has great advantages, allowing for drastically shortened trial times.

### 6.1. Metformin

Metformin is considered the first-line treatment for T2DM since the early 2000s, according to the guidelines of the International Diabetes Foundation (IDF Clinical Guidelines Task Force, 2006), but it is currently also considered a prime candidate for PD therapy. It inhibits hepatic gluconeogenesis, increases peripheral insulin sensitivity and glucagon-like peptide 1 (GLP-1) secretion, promoting peripheral glucose uptake, and reducing its bowel absorption at the same time. The mechanism of action is thought to be mainly through the inhibition of complex I of the mitochondrial transport chain, lowering ATP levels and indirectly activating the cellular metabolic state sensor AMPK (Zhou et al., 2016; Bahne et al., 2018; Baker et al., 2021; Drzewoski and Hanefeld, 2021). This increases fatty acid oxidation, reduces ROS production, and inhibits mTORC1 activating autophagic pathways and lysosomal biogenesis (Amin et al., 2019; Ma et al., 2023). Metformin also activates several downstream interactors including Bcl-1, CREB and PGC1, that promote cell viability and rescue mitochondrial defects, increasing their mass and improving their function (Kang et al., 2017; de Marañón et al., 2022). Finally, by modulation of Nrf2, FoxO3 and NF-κB activity, it exerts a protective effect in astrocytes and microglia (Ryu et al., 2018, 2020; Zhou et al., 2021).

In recent years, metformin has shown neuroprotective effects in both *in vitro* and *in vivo* models of PD, in the latter case by decreasing the loss of dopaminergic neurons and reducing motor symptoms, countering phospho-Ser129-aS aggregation in several ways. Pérez-Revuelta and colleagues have observed that metformin promotes the activity of PP2A, an enzyme of primary importance in reducing intracellular levels of phospho-Ser129-aS (Pérez-Revuelta et al., 2014; Greco et al., 2021); PP2A activity has indeed been shown to inhibit mTOR, promoting autophagy. Experiments in *C. elegans* and murine models of PD have both confirmed the effect of metformin in lowering phospho-Ser129-aS levels through an evolutionarily conserved mechanism (Pérez-Revuelta et al., 2014; Katila et al., 2017; Saewanee et al., 2021). Finally, metformin shows anti-glycating properties, acting as a scavenger of the aldehydic part of MG, preventing the accumulation of AGEs in subjects with T2DM (Kinsky et al., 2016).

### 6.2. GLP-1 agonists

Secreted by intestinal cells in response to food intake, especially carbohydrates, GLP-1 acts on several organs regulating glucose homeostasis. It stimulates insulin secretion from pancreatic β-cells in a glucose-dependent manner, reduces the secretion of glucagon, slows down gastric emptying, reduces the perception of hunger and thirst, and enhances peripheral glucose uptake (Hira et al., 2021).

Studies have shown that GLP-1 can inhibit mTOR stimulating autophagy in pancreatic β-cells and neurons. The binding of GLP-1 with its ubiquitously expressed membrane receptor GLP-1R triggers activation of the PI3K/AKT pathway. Subsequently, several downstream actors such as GSK3-β, FoxO1, NF-κB and Nrf2 exert cytoprotective effects (Buteau et al., 2006; Dai et al., 2013; Park et al., 2018; Costantino and Paneni, 2019; Li et al., 2020).

Interestingly, GLP-1R presence has been observed in the dopaminergic neuron of the encephalic area postrema, known for its role in modulating autonomic and reward responses, but also addiction mechanisms. GLP-1 seems therefore to be involved, at least in this specific region of the nervous system, in the modulation of dopamine synthesis and release (Yamamoto et al., 2003; Jensen et al., 2020).

Among the most important GLP-1R artificial agonists are exenatide, liraglutide, lixisenatide, semaglutide and NLY01. This class of molecules, found to be neuroprotective in mouse models treated with 6-OHDA or MPTP, is now a subject of interest for human use (Aviles-Olmos et al., 2013; Aslan et al., 2014; Jalewa et al., 2017). Promising results have been observed, with early-PD patients responding better to the treatment and showing motor and cognitive improvements (Athauda et al., 2019).

### 6.3. Glitazones

Glitazones, which includes pioglitazone, lobeglitazone and rosiglitazone are a class of oral antidiabetic drugs used in patients where first-line therapies fail to achieve glycemic control. Exerting their effect as agonists of the peroxisome proliferator-activated receptor PPARγ, a transcription factor of the nuclear ligand-activated receptor family, they increase tissues’ insulin sensitivity (Nanjan et al., 2018).

PPARγ induces the expression of a plethora of genes related to carbohydrate and lipid metabolism; at an encephalic level, expressed mainly in regions controlling reward and movement mechanisms such as the basal ganglia it has neuroprotective, with antioxidant, anti-inflammatory and anti-apoptotic properties (Warden et al., 2016; Villapol, 2018; Sonne et al., 2023). These effects, also linked to the downstream NF-κB signaling pathway activation, appear to be able to attenuate cognitive decline in various neurological diseases and neurodegenerative conditions (Delerive et al., 1999; Behl et al., 2021).

In several *in vivo* studies in PD-induced mice models, PPARγ agonists have shown a protective effect on dopaminergic neurons, with an improvement in motor symptoms. This is probably due to an inhibition of MAO-B, COX-1, COX-2 and iNOS activity, which reduce inflammation and preserve mitochondrial function and morphology (Xing et al., 2007; Quinn et al., 2008; Barbiero et al., 2014).

### 6.4. Insulin

The neuromodulatory, neurotrophic, and neuroprotective properties of insulin on the encephalon are well-known, therefore, its use in neurodegenerative contexts presents several potential beneficial effects (Yang et al., 1981; Shah and Hausman, 1993; Shuaib et al., 1995). However, two problems have hindered this approach until recently: the poor permeability of the blood–brain barrier (BBB) to its passage (Margolis and Altszuler, 1967) and the effect on hematic glycemia when parenterally administered. Therefore, new formulations have been studied to circumvent this problem; the most promising is via inhalation which exploits the permeability of the cribriform plate, shortening drug delivery to the target site and allowing precise modulation of administration. Several *in vivo* studies on murine PD models have demonstrated the efficacy of the molecule in this form and its neuroprotective activity (Fine et al., 2020; Iravanpour et al., 2021). Rajasekar and colleagues have shown how the use of intranasal insulin in mice treated with streptozocin was able to improve insulin-signaling cascade and attenuate neuroinflammation, neuronal loss, and protein aggregation. This was explained to be due to the modulation of NF-κB and PI3K/Akt pathway (Rajasekar et al., 2017).

Indeed, when intranasal insulin is administered, a canonical activation of PI3K/AKT and MAPK pathways is observed. From a neuroprotective perspective, there is an increased hypoxia-inducible factor-1 (HIF-1) activity, resulting in angiogenesis and endothelial proliferation (Zelzer, 1998). Furthermore, altered insulin signalling is associated with reduced mitochondrial fusion processes at the expense of organelle fission, resulting in increased ROS and reduced ATP levels (Kelley et al., 2002; Bach et al., 2003; Jheng et al., 2012).

Ruegsegger and colleagues demonstrated the ability of intranasal insulin to counteract these processes through the observation of an increase in the MFN1, MFN2, and OPA1 proteins, and a decrease in the DRP1 protein, involved in mitochondrial fusion and fission, respectively, (Ruegsegger et al., 2019a). Moreover, insulin also regulates mitochondrial quality control mechanisms by restoring the compromised activity of PINK1 and DJ-1. This appears particularly beneficial in brain areas of high mitochondrial activity such as the hypothalamus, and hippocampus (Onphachanh et al., 2017; Ruegsegger et al., 2019a; Su et al., 2020). Finally, insulin contributes to the removal of aggregates by promoting PP2A activity, which inhibits mTOR1 and dephosphorylates ULK1, initiating the autophagic process (Axe et al., 2008).

A 2018 clinical interventional study conducted at the University of Massachusetts on 15 patients for 4 weeks, involving intranasal administration of insulin, showed encouraging results. In this study, an improvement in motor performance, visuospatial memory, and verbal fluency was observed in PD subjects compared to placebo, due to a better ability to draw on mnemonic data (Novak et al., 2019).

## 7. Conclusion

To date, multiple pieces of evidence link T2DM to the onset of PD and other proteinopathies. The absence of cures for such medical conditions and the expected increase in their incidence in the coming years is driving clinical research. Hyperglycemia and insulin resistance, with the resulting protein aggregation, oxidative stress, and mitochondria dysfunction represent important and promising common medical targets, justifying interest in antidiabetic drugs, albeit the apparently different clinical settings. A large body of evidence on the efficacy of such drugs on pathways known to be involved in proteostasis and disrupted in PD exists. The data gathered in *in vivo* and *in vitro* models, together with the results of clinical trials leads to the view that the use of antidiabetic drugs, in combination with the current symptomatic medications is extremely encouraging.

## Author contributions

MG and AM conceptualized and drafted the original article, then revised it once in its final form. DM wrote a section of the manuscript and prepared the graphical abstract. CC performed data curation. MM funded the publication process, contributed to conception and revised its final form. All authors contributed to the article and approved the submitted version.
